# Blood *cis*-eQTL Analysis Fails to Identify Novel Association Signals among Sub-Threshold Candidates from Genome-Wide Association Studies in Restless Legs Syndrome

**DOI:** 10.1371/journal.pone.0098092

**Published:** 2014-05-29

**Authors:** Eva C. Schulte, Katharina Schramm, Claudia Schurmann, Peter Lichtner, Christian Herder, Michael Roden, Christian Gieger, Annette Peters, Claudia Trenkwalder, Birgit Högl, Birgit Frauscher, Klaus Berger, Ingo Fietze, Nadine Gross, Karin Stiasny-Kolster, Wolfgang Oertel, Cornelius G. Bachmann, Walter Paulus, Alexander Zimprich, Henry Völzke, Ulf Schminke, Matthias Nauck, Thomas Illig, Thomas Meitinger, Bertram Müller-Myhsok, Holger Prokisch, Juliane Winkelmann

**Affiliations:** 1 Neurologische Klinik und Poliklinik, Klinikum rechts der Isar, Technische Universität München, Munich, Germany; 2 Institut für Humangenetik, Helmholtz Zentrum München, Munich, Germany; 3 Institut für Humangenetik, Klinikum rechts der Isar, Technische Universität München, Munich, Germany; 4 Interfaculty Institute for Genetics and Functional Genomics, Ernst-Moritz-Arndt Universität Greifswald, Greifswald, Germany; 5 Institute for Clinical Diabetology, German Diabetes Center, Leibniz Center for Diabetes Research at Heinrich Heine University Düsseldorf, Düsseldorf, Germany; 6 German Center for Diabetes Research (DZD e.V.), partner Düsseldorf, Düsseldorf, Germany; 7 University Clinics of Endocrinology and Diabetology, University Hospital Düsseldorf, Düsseldorf, Germany; 8 Institute for Genetic Epidemiology, Helmholtz Zentrum München, Munich, Germany; 9 Institute for Epidemiology II, Helmholtz Zentrum München, Munich, Germany; 10 Paracelsus Elena Klinik, Kassel, Germany; 11 Department of Neurosurgery, University Medical Center, Georg August Universität Göttingen, Göttingen, Germany; 12 Neurologische Klinik, Medizinische Universität Innsbruck, Innsbruck, Austria; 13 Institut für Epidemiologie und Sozialmedizin, Westfälische Wilhelms Universität Münster, Münster, Germany; 14 Zentrum für Schlafmedizin, Charite Universitätsmedizin, Berlin, Germany; 15 Neurologische Klinik, Philips Universität Marburg, Marburg, Germany; 16 Somnomar Institut für Medizinische Forschung und Schlafmedizin, Marburg, Germany; 17 Abteilung für Neurologie, Paracelsus Klinik Osnabrück, Osnabrück, Germany; 18 Department of Clinical Neurophysiology, University Medical Center, Georg August Universität Göttingen, Göttingen, Germany; 19 Neurologische Klinik, Medizinische Universität Wien, Vienna, Austria; 20 Institute for Community Medicine, University Medicine Greifswald, Greifswald, Germany; 21 Institute of Neurology, University Medicine Greifswald, Greifswald, Germany; 22 Institute of Clinical Chemistry and Laboratory Medicine, University Medicine Greifswald, Greifswald, Germany; 23 Research Unit of Molecular Epidemiology, Helmholtz Zentrum München, Munich, Germany; 24 Hannover Unified Biobank, Hannover Medical School, Hannover, Germany; 25 Max-Planck Institute for Psychiatry, Munich, Germany; 26 Munich Cluster for Systems Neurology (SyNergy), Munich, Germany; National Institutes of Health, United States of America

## Abstract

Restless legs syndrome (RLS) is a common neurologic disorder characterized by nightly dysesthesias affecting the legs primarily during periods of rest and relieved by movement. RLS is a complex genetic disease and susceptibility factors in six genomic regions have been identified by means of genome-wide association studies (GWAS). For some complex genetic traits, expression quantitative trait loci (eQTLs) are enriched among trait-associated single nucleotide polymorphisms (SNPs). With the aim of identifying new genetic susceptibility factors for RLS, we assessed the 332 best-associated SNPs from the genome-wide phase of the to date largest RLS GWAS for *cis*-eQTL effects in peripheral blood from individuals of European descent. In 740 individuals belonging to the KORA general population cohort, 52 *cis*-eQTLs with p_nominal_<10^−3^ were identified, while in 976 individuals belonging to the SHIP-TREND general population study 53 *cis*-eQTLs with p_nominal_<10^−3^ were present. 23 of these *cis*-eQTLs overlapped between the two cohorts. Subsequently, the twelve of the 23 *cis*-eQTL SNPs, which were not located at an already published RLS-associated locus, were tested for association in 2449 RLS cases and 1462 controls. The top SNP, located in the *DET1* gene, was nominally significant (p<0.05) but did not withstand correction for multiple testing (p = 0.42). Although a similar approach has been used successfully with regard to other complex diseases, we were unable to identify new genetic susceptibility factor for RLS by adding this novel level of functional assessment to RLS GWAS data.

## Introduction

Restless legs syndrome (RLS) is a common sensory-motor disorder characterized by dysesthesias affecting the legs, triggered by periods of rest, relieved by movement and occurring mostly during the evening and at night. [1] Consequences are severe sleep disturbances, depression, anxiety and possibly also increased cardiovascular risk. [2], [3] RLS is a complex polygenic phenotype and genome-wide association studies (GWAS) have identified a total of six genomic loci associated with the disease. [4]–[7] Still, the susceptibility alleles known to date only explain about 6.8% of the total heritability [6]. It is likely that additional risk loci of weaker effect sizes exist that have not yet been ascertained in the GWAS.

It has been shown that single nucleotide polymorphisms (SNPs) associated with complex genetic traits are more likely to have an effect on gene expression and, thus, represent expression quantitative trait loci (eQTLs). [8], [9] The use of *cis*-eQTL analyses in prioritizing sub-threshold association signals for GWAS follow-up, has already been successfully employed with regard to several complex diseases such as Crohn’s disease [10], asthma [11], or schizophrenia [12]. Accordingly, we sought to prioritize sub-threshold RLS association signals from an RLS GWAS [6] via *cis*-eQTLs in the human blood for follow-up association study seeking to highlight additional genetic factors involved in RLS.

## Materials and Methods

### Ethics Statement and Data Availability

The KORA and SHIP-TREND studies as well as the recruitment of the RLS case/control sample was carried out in accordance with the recommendations of the Declaration of Helsinki and was approved by ethics committees of the “Bayerische Landesärztekammer” and the Technische Universität München (for KORA and the RLS case/control sample) and the University of Greifswald (for SHIP-TREND). Written informed consent was obtained from each of the study participants. Due to ethics constraints, full expression and genotyping data sets cannot be made available to the general public. However, interested researches can apply for access to all data (KORA: http://www.helmholtz-muenchen.de/en/kora-en/information-for-scientists/contact-persons/index.html; SHIP: http://www.medizin.uni-greifswald.de/cm/fv/english/ship_en.html).

### Study Design and SNP Selection

**Figure 1:**
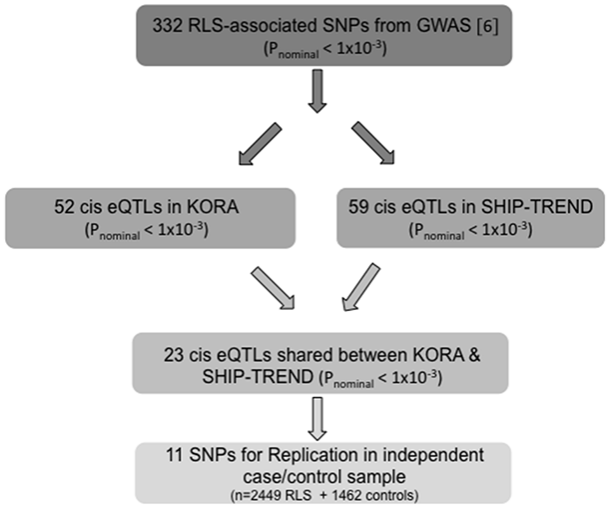
Study Design.

The objective of the study was to use blood-based *cis*-eQTL analysis as a filter in the identification of new RLS susceptibility factors from sub-threshold association signals from a previously published GWAS. We selected all SNPs with an association signal of p_nominal_<1×10^−3^ (λ-corrected, n = 332) from a recently published RLS GWAS [7] for *cis*-eQTL analysis to identify SNPs linked to differential mRNA expression (*cis*-eSNPs). These 332 SNPs represented 197 loci containing a single SNP and 101 loci with two or more SNPs in very high linkage disequilibrium (LD; r^2^≥0.8). *cis*-eQTLs based on all 332 SNPs were identified in 740 individuals belonging to the KORA general population-based study and, in parallel, in 976 individuals belonging to the SHIP-TREND general population-based study. The *cis*-eSNPs with p_nominal_<1×10^−3^ present in both cohorts and not located at loci of published association with RLS [4]–[7] were replicated in an independent case/control sample (Figure 1) with the objective of identifying new RLS-associated SNPs.

### Cohorts and Case/Control Samples

#### KORA F4 cohort (discovery sample 1)

Based in southwestern Germany, KORA (Cooperative Health Research in the Region of Augsburg) is a regional research platform for population-based surveys and follow-up studies. Whole-blood samples used for expression analysis in this study were collected from 740 subjects aged 62 to 81 years as part of the KORA F4 survey between 2006 and 2008 [13], [14].

#### SHIP-TREND cohort (discovery sample 2)

SHIP (Study of Health in Pomerania in northeastern Germany) is a population-based project consisting of two independent cohorts, SHIP and SHIP-TREND [15], [16]. For eQTL analysis, the SHIP-TREND cohort was used where a total of 976 samples, from individuals aged 20 to 81 years, with both imputed genotypes and whole-blood gene expression data were available [16].

#### Case/control sample for eSNP association study

The sample comprised 2449 German and Austrian individuals with RLS (average age 48.0±34.7 years, 70.7% female) and 1462 individuals belonging to the S4 survey of the KORA general population cohort [17] (average age 49.9±13.4 years, 51.7% female), who were not genotyped in the genome-wide phase of the GWAS [6]. Both case and control populations were entirely of European descent. In all patients, diagnosis of primary RLS was based on the diagnostic criteria of the International RLS Study Group [1] as assessed in a personal interview conducted by an RLS expert.

### Genome-wide Genotyping (Discovery Samples 1 and 2)

As described previously [16], [19], genome-wide genotyping of the KORA sample was performed on Affymetrix Genome-Wide Human SNP Arrays 6.0. SNPs with minor allele frequency (MAF) <5%, a call rate <98% or a significant deviation from Hardy-Weinberg Equilibrium (HWE) (p<1×10^−5^) were excluded. Genotyping of the SHIP-TREND cohort has been described in detail [16]. In short, participants of the SHIP-TREND cohorts were genotyped using Illumina HumanOmni2.5-Quad arrays. Arrays with a call rate below 94% as well as individuals with a mismatch between the reported and genotyped sex were excluded. Imputation of autosomal genotypes in the SHIP-TREND cohort was performed using IMPUTE v2.1.2.3 [18] against the 1000 genomes phase I (interim) reference panel released June 2011 (all ancestries panel, build 37).

### Gene Expression Data and Quality Control (Discovery Samples 1 and 2)

Sample selection and preparation of whole-blood gene expression analyses in KORA F4 and SHIP-TREND have been described [16], [19]. Briefly, in both studies blood was taken and stored in PAXgene blood RNA tubes, RNA was isolated using the PAXgene Blood miRNA Kit (Qiagen, Hilden, Germany) and reverse transcribed using the Illumina TotalPrep-96 RNA Amp Kit (Ambion, Darmstadt, Germany). The labelled cRNA was hybridized to Illumina HumanHT-12 v3 Expression BeadChip arrays and scanned using the Illumina Bead array reader. The GenomeStudio v2010.1 Gene Expression Module was used for quality control and to impute missing values. Subsequently, raw gene expression intensities were exported from Illumina’s GenomeStudio software to the R environment, where log2-transformation and quantile normalization where carried out. After quality control (exclusion of individuals with less than 6000 significantly detected probes (p<0.01) and individuals with a reported vs. calculated gender mismatch), 740 KORA F4 and 976 SHIP-TREND samples with gene expression and genotype data where available for analysis.

### Determination of cis- and Trans-eQTLS

The eQTL analysis was carried out for all 332 selected sub-threshold SNPs. For *cis*-eQTL analyses, all probes less than 500 kilobases (kb) away from the 332 selected SNP were used. *Trans*-eQTLs were determined for all probes more than 5 megabases (Mb) away from the SNP of interest.

Associations between the SNP and the respective mRNA probes were analyzed using a linear model with adjustment for age and sex. P-values were corrected using the Benjamini and Hochberg procedure.

### Genotyping of Replicated cis-eQTL SNPs in Case/Control Replication Sample

Genotyping was performed on the MassARRAY system using MALDI-TOF mass spectrometry with iPLEX Gold chemistry (Sequenom Inc, San Diego, CA, USA). Genotypes were called with SpectroTYPER 3.4. Genotype clustering was visually checked by an experienced evaluator. SNPs with a call rate <95%, MAF <5%, and Hardy-Weinberg p-value<1×10^−5^ in controls were excluded. Known RLS-associated SNPs were not followed up in the replication. Associations were tested using the allelic test as implemented in PLINK [20].

### Analysis of Brain-derived cis-eQTLs

The brain *cis*-eQTL status of all 23 replicated *cis*-eSNPs was analyzed using the NCBI GTEx eQTL browser (http://www.ncbi.nlm.nih.gov/gtex/GTEX2/gtex.cgi, accessed August 5, 2012; expression data from cerebellum, frontal and temporal cortex and pons, n = 142 to 144) [22] and the seeQTL browser (http://gbrowse.csbio.unc.edu/cgi-bin/gb2/gbrowse/seeqtl/, accessed August 5, 2012; expression data from whole brain, n = 193) [23].

## Results

### RLS-associated SNPs are more likely to be cis-eQTLs

To test whether RLS-associated SNPs are more commonly *cis*-eQTLs than those not associated, we compared the number of *cis*-eQTLs among the 332 most significantly associated SNPs (all with p_nominal_<1×10^−3^) from the latest RLS GWAS [6] to the 332 with the worst association p-values. MAF distribution was similar in both groups. Among the associated 332 SNPs, 52 *cis*-eQTLs (p_nominal_<1×10^−3^) were found while 37 *cis*-eQTLs were present among the 332 not-associated SNPs. After very stringent LD pruning (setting a threshold of r^2^≤0.5), which was necessary as there was significantly higher LD among the potentially associated SNPs compared to the not associated SNPs, we found evidence for an enrichment of *cis*-eQTLs (p_nominal_<1×10^−3^) in the associated vs. the not-associated SNPs (34 *cis*-eQTLs among 246 SNPs harboring the most significant association signals vs. 28 *cis*-eQTLs among 313 SNPs showing the least significant association signals; Fisher’s exact test, one-sided, p<0.05, OR = 1.63).

### Analysis of Specific cis-eQTLs

Among the 332 best-associated SNPs, 52 *cis*-eSNPs with p_nominal_<1×10^−3^ resulting in 45 independent *cis*-eQTLs were found when assessed in whole-blood samples from 740 KORA F4 controls. These 45 *cis*-eQTLs represent 33 loci with LD<0.8. Four SNPs (intronic rs17487827 in *BARD1* as well as intronic rs6714954, rs7592599 and rs13387588 in *SLC4A5*) represented *cis*-eQTLs of transcriptome-wide significance (p_nominal_<1×10^−8^) (Table 1). In the 976 SHIP-TREND samples, 59 *cis*-eSNPs with p_nominal_<1×10^−3^ resulting in 46 independent *cis*-eQTLs and 29 independent loci were identified (Table 1).

**Table 1:** RLS-associated SNPs representing *cis*-eQTLs in peripheral blood.

SNP	Locted in	Gene expression altered	Association(p_nominal_ frompast GWAS) [7]	KORA*cis*-eQTL(p_nominal_)	KORAmajorallele	KORAbeta	SHIP-TREND*cis*-eQTL(p_nominal_)	SHIP-TRENDmajorallele	SHIP-TRENDbeta	AssociationReplication(p_nominal_)	AssociationReplication(p_corrected_)	
**rs17487827**	***BARD1***	***BARD1***	**0.00021**	**1.02E-13**	**C**	**−0.171**	**9.20E-17**	**C**	**−0.110**	**0.255**	**NS**	
**rs6746899**	***intergenic***	***intergenic***	**0.00089**	**1.53E-08**	**A**	**−0.172**	**1.61E-08**	**A**	**−0.130**	**no assay**		
**rs6714954**	***NBC4***	***MRPL53/AUP1***	**0.00021**	**2.22E-08**	**G**	−0.145	**8.50E-07**	**G**	**0.104**	**0.148**	**NS**	
**rs9920066**	***DET1***	***DET1***	**0.00053**	**2.26E-08**	**T**	**−0.071**	**2.31E-08**	**T**	**−0.054**	**0.038**	**NS (0.418)**	
**rs9354792**	***CR595314***	***CR595314***	**0.00078**	**3.20E-08**	**A**	**0.086**	**3.36E-10**	**A**	**0.048**	**0.521**	**NS**	
**rs17125761**	***intergenic***	***ERO1L***	**0.00054**	**9.40E-07**	**T**	**−0.091**	**4.85E-05**	**T**	**0.066**	**0.965**	**NS**	
**rs11024433**	***SERGEF***	***SAAL1***	**0.00088**	**5.58E-06**	**G**	**−0.074**	**1.35E-06**	**G**	**0.051**	**0.557**	**NS**	
**rs7670748**	***intergenic***	***intergenic***	**0.00013**	**7.86E-06**	**C**	**0.134**	**8.78E-12**	**C**	**0.125**	**0.499**	**NS**	
**rs2029361**	***intergenic***	***SMC4***	**0.00095**	**9.95E-06**	**C**	**0.099**	**0.0003**	**C**	**0.063**	**0.385**	**NS**	
**rs738415**	***intergenic***	***BC033837***	**0.00062**	**1.04E-05**	**G**	**0.142**	**1.49E-07**	**G**	**−0.119**	**0.937**	**NS**	
**rs4388643**	***ZNF364***	***ANKRD35***	**0.00047**	**1.51E-05**	**G**	**0.072**	**4.41E-06**	**G**	**−0.055**	**0.533**	**NS**	
**rs28670272**	***MAP2K5***	***CALML4***	**0.00025**	**0.00019**	**A**	**−0.064**	**4.62E-06**	**A**	**0.053**			
rs28670272	*MAP2K5/LBXCOR1*	*CALML4*	0.00025	1.93E-04	**A**	−0.064	4.62E-06	**A**	−0.053			
**rs12593813**	***MAP2K5***	***CALML4/MAP2K5***	**1.49E-06**	**0.00022**	**G**	**−0.059**	**3.65E-06**	**G**	**0.048**			
rs12593813	*MAP2K5*	*CALML4*	**1.49E-06**	0.00022	**G**	−0.058	3.65E-06	**G**	0.048			
rs11635424	*MAP2K5*	*CALML4*	1.52E-06	0.00022	**G**	−0.058	3.59E-06	**G**	0.049			
rs868037	*MAP2K5*	*CALML4*	9.51E-07	0.00026	**G**	−0.058	2.62E-06	**G**	0.049			
**rs683856**	***KCNC3***	***NAPSB***	**0.00018**	**0.00034**	**T**	**−0.125**	**1.64E-07**	**T**	**0.116**	**0.618**	**NS**	
rs4489954	*MAP2K5*	*CALML4*	8.26E-06	0.00038	**G**	−0.057	1.32E-06	**G**	−0.052			
rs1026732	*MAP2K5*	*CALML4*	4.01E-06	0.00051	**G**	−0.055	3.56E-06	**G**	0.048			
rs6494696	*MAP2K5/LBXCOR1*	*CALML4*	3.70E-06	0.00042	**G**	−0.056	3.23E-06	**G**	0.049			
rs4489954	*MAP2K5*	*MAP2K5*	8.26E-06	0.00044	**G**	−0.057	0.00083	**G**	−0.035			
rs28670272	*MAP2K5/LBXCOR1*	*MAP2K5*	0.00025	0.00098	**A**	−0.049	7.13E-05	**A**	−0.045			

A total of 23 *cis*-eQTLs were found in both KORA F4 and SHIP-TREND at p_nominal_<1×10^−3^. SNPs which were carried into the replication phase are printed in bold. NS = not significant.

Of the six known RLS loci [5], [6], [7], [8], only SNPs located on chromosome15q were *cis*-eSNPs with p_nominal_<1×10^−3^ in both cohorts (Table 1). The expression change seen, however, did not affect the primary candidate genes at these loci but rather another gene in the vicinity.

Of the identified *cis*-SNPs, 23 overlapped between the KORA and SHIP-TREND samples and eleven of these reached transcriptome-wide significance in either cohort but only rs17487827 in *BARD1* reached transcriptome-wide significance independently in both cohorts. The 23 replicated *cis*-eSNPs contained nine that were dependent upon SNPs at the known RLS-associated locus on chromosome *15q* (*MAP2K5/SKOR1*). None of these were associated with altered gene expression levels of *SKOR1* and only two *cis*-eSNPs (rs4489954 and rs28670272) affected the gene expression levels of *MAP2K5*, the two candidate genes underlying the GWAS association signal at this locus. Instead, seven *cis*-eSNPs coincided with differential expression of *CALML4*, located approximately 400 kb upstream of the known locus.

The remaining 14 *cis*-eSNPs represented twelve individual loci as three SNPs (rs7592599, rs6714954 and rs13387588) located in *SLC4A5* all associated with decreased expression levels of two neighboring genes, *AUP1* and *MRPL53*. Directions of differential expression concurred in 42.9% (6 out of 14) of *cis*-eSNPs in the two cohorts (Table 1).

### Trans-eQTLs Linked to RLS-associated SNPs

We also assessed transcriptome-wide *trans*-eQTLs in the whole-blood samples for 13 SNPs known to be associated with RLS [4]–[7]. However, none of the *trans*-eQTLs identified in KORA F4 or SHIP-TREND were also found at p_nominal_<1×10^−3^ in the respective other cohort (data not shown).

### Replication of Sub-threshold SNPs Representing cis-eQTLs

Twelve *cis*-eSNPs with p_nominal_<10^−3^ in both the KORA F4 and the SHIP-TREND study were selected for replication in an independent sample comprising 2449 German and Austrian RLS cases and 1462 KORA general population-based controls. Due to technical reasons, intergenic SNP rs6746899 could not be included in the replication. One SNP in *DET1* (rs9920066) showed nominally significant association (p_nominal_<0.05) but did not withstand Bonferroni correction (p_corrected_ = 0.42) while the other ten SNPs were not associated with the RLS phenotype in the replication sample (Table1).

### Expression in Brain

The relevance of blood-based *cis*-eQTLs or *cis*-eSNPs to neurologic and psychiatric diseases has been shown. [9] However, differences between blood eQTLs or eSNPs and brain-based eQTLs or eSNPs have also been demonstrated. [23] Therefore, we analyzed the brain *cis*-eQTL status of all 23 *cis*-eSNPs seen in both general population cohorts using the NCBI GTEx eQTL and the seeQTL browsers. None of the 23 blood *cis*-eSNPs were also *cis*-eSNPs with p_nominal_<1×10^−3^ in the cerebellum, frontal and temporal cortex or pons (n = 142 to 144) [22] or in whole brain (n = 193) [23].

## Discussion

Blood *cis*-eQTL analysis has been successfully used in enhancing output from GWAS. [10], [11], [12] Here, we evaluated *cis*-eSNPs and *cis*-eQTLs linked to (potential) RLS susceptibility genes identified in previous RLS GWAS in order to prioritize sub-threshold candidates for follow-up evaluation.

Apart from one SNP in the de-etiolated 1 encoding gene *DET1* (rs9920066) that reached nominal significance in the replication phase but did not withstand correction for multiple testing, we did not identify any novel susceptibility factors for RLS.

Next to the possibility that eQTLs in general participate very little in bringing about the RLS phenotype, it is possible that our study lacks the power to establish an association between the RLS phenotype and the SNPs underlying relevant eQTLs. Statistical power calculation using the Purcell Power Calculator [24] revealed that in order to replicate an association for one SNP such as, for example, *DET1* rs9920066 (OR = 1.11 (95% confidence interval: 1.00–1.22)), with MAF = 0.30 at α  = 0.05, one would need a minimum of 5,767 cases and 5,767 controls to achieve 80% power.

Another caveat has to be that *cis*-eQTLs employed in selecting SNPs for replication were evaluated in peripheral blood and not a more disease-specific tissue. Although the underlying pathophysiology is not entirely clear, an involvement of the central nervous system in RLS pathophysiology seems likely. Evaluation of the 23 common *cis*-eSNPs in two human brain expression data sets showed that none of the blood *cis*-eQTLs were also found in the brain. Whether this is due to the smaller number of samples (347 brain samples vs. 1716 blood samples) or the fact that *cis*-eQTLs dependent on (potentially) RLS-associated SNPs in the peripheral blood do not overlap with those in the brain and are not functionally relevant for disease pathogenesis, remains unclear. It is known that eQTLs can be specific to developmental time points [22], [23] and brain regions [21] and that they were, therefore, not detected in the available data. In this context, an RLS-linked common variant was recently shown to alter gene expression in the murine ganglionic eminences, the primordial basal ganglia, during development [25]. Accordingly, it will be of great interest to evaluate eQTLs in specifically this neuroantomic region and at this developmental time point in the future.

Although none of the RLS-associated SNPs selected for follow-up could be replicated, two additional interesting aspects emerged. Firstly, one of the known RLS susceptibility loci on chromosome 15q [4]–[6] comprising RLS candidate genes *MAP2K5* and *SKOR1* harbored nine *cis*-eSNPs with p_nominal_<1×10^−3^. Two of these showed altered *MAP2K5* expression dependent on the RLS-risk allele though in different directions in KORA F4 and SHIP-TREND, while none were related to altered expression of *SKOR1*. Interestingly, seven RLS-linked SNPs in *MAP2K5* were further related to altered expression of calmodulin-like 4 (*CALML4*), a gene located approximately 400 kb upstream of the RLS-associated *MAP2K5/SKOR1* locus. However, here, too, the direction of differential expression was not the same in both cohorts. Despite the fact that several studies have been successful in using *cis*-eQTLs to fine-map or provide functional support for specific genes at a GWAS locus [11], [26], [27], in our study, the situation is not as clear. It is possible that potential RLS-associated expression changes in *CALML4* are due to SNPs in *CALML4,* which are in high LD with RLS-associated SNPs at the *MAP2K5/SKOR1* locus. Alternatively, it cannot be excluded that variation in *CALML4* instead of, or in addition to, *MAP2K5/SKOR1* could play a role in RLS pathogenesis, as has been postulated in other complex traits such as the body mass index [28] or that these expression changes are artificial as they do not concur in the two cohorts.

Overall, we were unable to establish a new genetic susceptibility factor for RLS, although, at least in the case of *DET1*, this may be due to the lack of power to replicate alleles conferring only a small risk increase. Our study is challenged by the fact that *cis*-eQTLs were evaluated in peripheral blood and not a tissue of more pathophysiologic relevance to RLS. In the future, as the neuroanatomic correlates of RLS become more defined and more expression profiles of different brain regions become available, it will be interesting to assess whether the blood *cis*-eQTLs also play a role in brain-region-specific, RLS-allele-dependent eQTLs and in disease development.
